# Alpha‐lipoic acid downregulates TRPV1 receptor via NF‐κB and attenuates neuropathic pain in rats with diabetes

**DOI:** 10.1111/cns.13303

**Published:** 2020-03-16

**Authors:** Bing‐Yu Zhang, Yi‐Lian Zhang, Qian Sun, Ping‐An Zhang, Xi‐Xi Wang, Guang‐Yin Xu, Ji Hu, Hong‐Hong Zhang

**Affiliations:** ^1^ Department of Endocrinology The Second Affiliated Hospital Soochow University Suzhou China; ^2^ Center for Translational Pain Medicine Institute of Neuroscience Soochow University Suzhou China

**Keywords:** dorsal root ganglion, NF‐κB, painful diabetic neuropathy, transient receptor potential vanilloid‐1

## Abstract

**Aims:**

Painful diabetic neuropathy (PDN) is a refractory complication of diabetes. The study aimed to investigate the role of α‐lipoic acid (ALA) on the regulation of transient receptor potential vanilloid‐1 (TRPV1) in dorsal root ganglion (DRG) neurons of rats with diabetes.

**Methods:**

Whole‐cell patch‐clamp recordings were employed to measure neuronal excitability in DiI‐labeled DRG neurons of control and streptozotocin (STZ)‐induced diabetic rats. Western blotting and immunofluorescence assays were used to determine the expression and location of NF‐κBp65 and TRPV1.

**Results:**

STZ‐induced hindpaw pain hypersensitivity and neuronal excitability in L4‐6 DRG neurons were attenuated by intraperitoneal injection with ALA once a day lasted for one week. TRPV1 expression was enhanced in L4‐6 DRGs of diabetic rats compared with age‐matched control rats, which was also suppressed by ALA treatment. In addition, TRPV1 and p65 colocated in the same DRG neurons. The expression of p65 was upregulated in L4‐6 DRGs of diabetic rats. Inhibition of p65 signaling using recombinant lentiviral vectors designated as LV‐NF‐κBp65 siRNA remarkably suppressed TRPV1 expression. Finally, p65 expression was downregulated by ALA treatment.

**Conclusion:**

Our findings demonstrated that ALA may alleviate neuropathic pain in diabetes by regulating TRPV1 expression via affecting NF‐κB.

## 1 INTRODUCTION

Diabetic peripheral neuropathy is one of the most common chronic complications of diabetes, occurring in 54%‐59% patients with type 1 diabetes and 45%‐70% patients with type 2 diabetes.^1^, ^2^ About a third of patients with diabetic peripheral neuropathy suffer aberrant pain sensation, including spontaneous pain, hyperalgesia, and allodynia.^3^, ^4^ The mechanisms of diabetic painful neuropathy (DPN) have not been completely defined, and the pharmacological treatment remains a challenge for all the physicians. Although several analgesic drugs, such as duloxetine, pregabalin, and opioid, have been used in clinical practice, they are not effective for all patients. We need more evidences on the effectiveness of other drugs in the treatment of painful diabetic neuropathy.

Transient receptor potential vanilloid‐1 (TRPV1) is a ligand‐gated nonselective cation channel. It is widely distributed in the peripheral and central nervous systems. In dorsal root ganglion (DRG), TRPV1 is mainly distributed on medium‐ and small‐sized nociceptive neurons. When the receptor is activated, a large amount of Ca^2+^ influx promotes the forming of pain sensitivity. Studies have shown that the receptor plays an important role in mediating various pains like diabetic neuropathic pain.^5^ So, TRPV1 is an important pain‐related gene and analgesic target.

NF‐κB is a well‐known transcription factor. It is involved in multiple interactions such as oxidative stress, inflammation, and regulation of pain‐related key molecule 6, 7, 8 and plays an important role in chronic complications of diabetes.^9^, ^10^ Our previous study reported that NF‐κB specifically bound to the P2X3 promoter region and promoted P2X3 receptor expression, contributing to diabetic neuropathic pain.^8^ At present, it has not been reported whether NF‐κB could regulate TRPV1 expression.

Alpha‐lipoic acid (ALA), one of the first‐choice drugs for the treatment of diabetic peripheral neuropathy, has been widely recognized. ALA is a coenzyme found in mitochondria and acts as a cofactor for many mitochondrial enzymes.^11^ ALA contains a disulfide structure and has significant electrophilicity and the ability to react with free radicals, so it has strong oxidation resistance. Its functions include the following: (a) removal of ROS, (b) regeneration of endogenous and exogenous antioxidants such as glutathione (GSH) and vitamins C and E, (c) repair of oxidized proteins, (d) metal chelation, and (e) regulation of gene transcription factor.^12^ Growing studies have shown that ALA has beneficial effects on disease characterized by increased oxidative stress, such as diabetes, and its complications.^12^ However, as stated in the treatment guideline,^13^ there is insufficient evidence to determine whether ALA is effective for the treatment of PDN. In this study, we studied the analgesic effect of ALA in diabetic painful neuropathy and its possible regulation of NF‐κB and TRPV1.

## 2 METHODS AND MATERIALS

### 2.1 Induction of diabetes

We use adult female Sprague Dawley rats weighted 180‐200g throughout the whole experiments. We induce diabetic rats by a single intraperitoneal injection of STZ (65 mg/kg i.p.; Sigma‐Aldrich), which was dissolved in freshly prepared citrate buffer (10 mmol/L, Na citrate, pH 4.3‐4.4). Correspondingly, the control group was treated with an equivalent volume of citrate buffer. One week later, diabetes was confirmed by glucometer (Johnson & Johnson) in blood samples that were obtained from the tail vein. Only rats with a blood glucose concentration > 15.0mmol/L were reckoned as the exactly diabetic ones and were used.

### 2.2 Measurement of hindpaw withdrawal threshold

We perform behavioral experiments on diabetic rats and age‐matched control rats. Hindpaw withdrawal threshold was measured before and after the injection of STZ or citrate buffer. We measure the rat paw withdrawal threshold (PWT) and thermal paw withdrawal latency (PWL) as described previously.^8^, ^14^ All the behavioral experiments were performed under blind conditions.

### 2.3 Whole‐cell patch‐clamp recordings

The excitability of DRG neurons specific‐innervating the hindpaw was measured using whole‐cell patch‐clamp recording techniques, as described previously.^15^ Small‐ and medium‐sized DRG neurons were chosen in our study. The pipette solution contained (in mmol/L) 140 potassium gluconate, 10 HEPES, 10 NaCl, 10 glucose, 5 EGTA, and 1 CaCl_2_ (pH = 7.25, adjusted with KOH; osmolarity: 292 mOsm/kg·H_2_O). The external solution contained (in mmol/L) 130 NaCl, 2 KH_2_PO_4_, 5 KCl, 1 MgCl_2,_ 2.5 CaCl_2_, 10 HEPES, and 10 glucose (pH = 7.2, adjusted with NaOH, osmolarity: 295‐300 mOsm/kg·H_2_O). The voltage was clamped at –60 mV. Whole‐cell voltage and current were recorded with a HEKA EPC10 patch‐clamp amplifier. The data were obtained and analyzed by Fit Master from HEKA. Cells were characterized by cell membrane capacitance (Cm), input resistance (Rin), resting membrane potential (RP), rheobase, action potential (AP) threshold, AP overshoot, and duration elicited by current stimulation. The frequency of APs stimulated by 2× and 3× rheobase and ramp current stimulation (300, 500 pA/sec ramp current) was recorded under the current‐clamp mode.

### 2.4 Western blotting analysis

The expressions of p65 and TRPV1 in L4–L6 DRGs from control and diabetic rats were detected using Western blotting analysis. Anti‐TRPV1 (#ACC‐030, Alomone, 1:200), anti‐p65 (#6956, Cell Signaling Technology, 1:1000), and corresponding horseradish peroxidase‐conjugated secondary anti‐rabbit and anti‐mouse antibodies at dilutions of 1:2000 and 1:2000 were used to probe the proteins, respectively. We analyze the densities of protein bands by NIHA ImageJ software.

### 2.5 Lentivirus vector generation and intrathecal injection

The siRNA targeting the cDNA sequence of rat p65 (GenBank Accession #NM_199267) was 5‐GCAGUUCGAUGCUGAUGAAUU‐3. A scrambled sequence was designed as a negative control (NC). The replication‐deficient, self‐inactivating LV pFU‐GWRNAi‐GFP (Shanghai Genechem Co., Ltd.) was generated. The cDNAs relative to the p65 siRNA and NC were subcloned into the vector pFU‐GW‐RNAi‐GFP. The resulting recombinant LVs were designated as LV‐p65 siRNA or LV‐NC. The final titer of LV‐p65 siRNA and LV‐NC was 1 × 10^9^ transduction units (TU)/mL. We injected the lentiviruses into rats intrathecally.

### 2.6 Immunofluorescence assay

Immunofluorescence assay was performed as the previous reports.^8^ Mouse anti‐p65 (#6956, Cell Signaling Technology, 1:50) and rabbit anti‐TRPV1 (#ACC‐030, Alomone, 1:50) were used in this study. The labeled neurons were quantified manually via using NIH ImageJ software.

### 2.7 Data analysis

All data from the studies were expressed as mean ± SEM. Software MATLAB (Math Works) and Origin Pro 8 (Origin Lab) were used for data analysis. Significance was determined using a two‐sample *t* test, Mann‐Whitney *U* test, Dunn post hoc test following Friedman ANOVA, and two‐way repeated‐measures ANOVA followed by Tukey post hoc test. A *P* value < .05 was considered to be statistically significant.

## 3 RESULTS

### 3.1 ALA treatment attenuated mechanical allodynia and thermal hyperalgesia in diabetic rats

**Figure 1:**
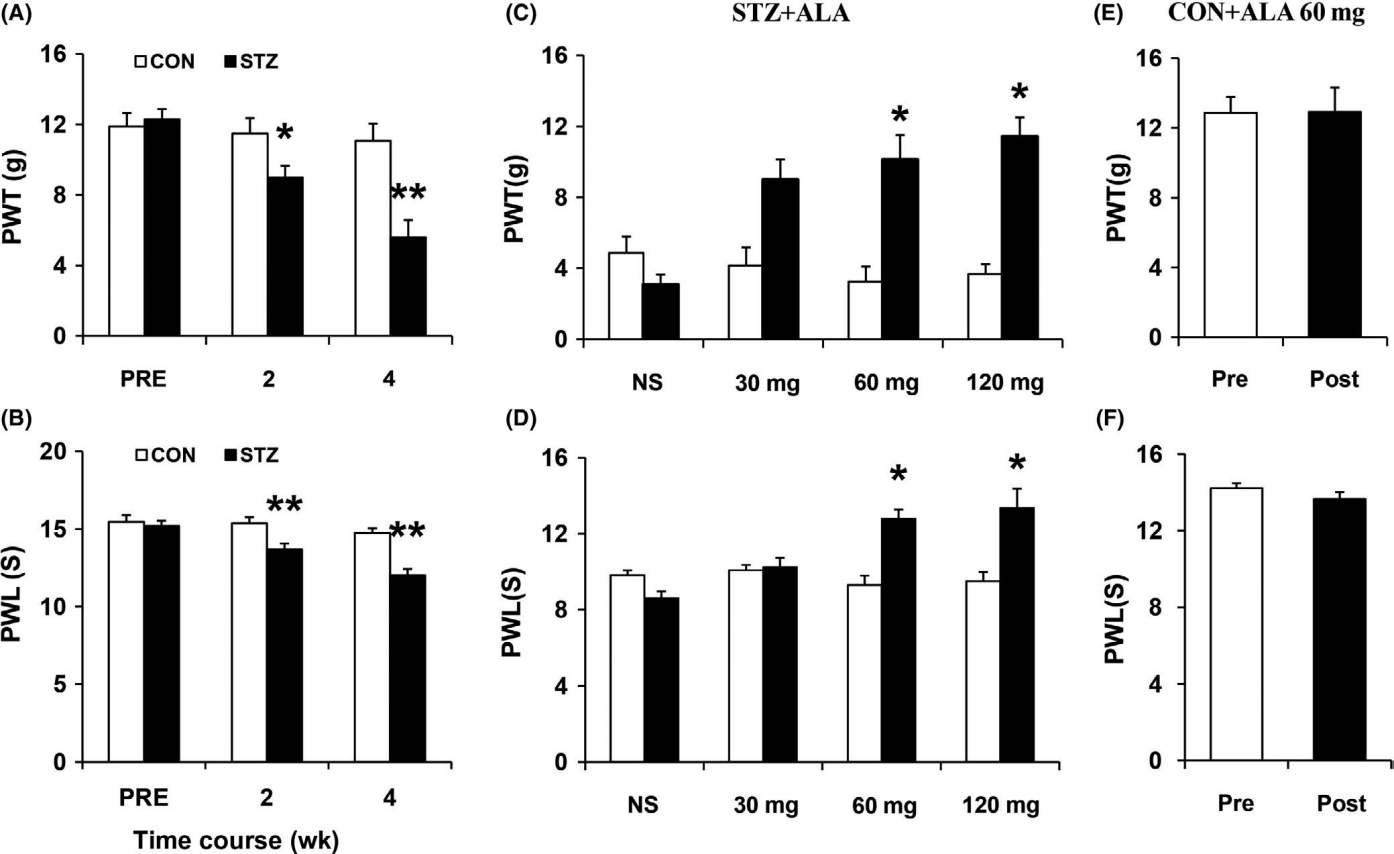
ALA treatment attenuated STZ‐induced mechanical allodynia and thermal hyperalgesia in rats with diabetes. A, The mechanical PWT began to decrease from 2 wk after STZ injection and became lower at the 4th weeks (N = 10 for each group, *P < .05, **P < .01, compared with CNT group, Friedman ANOVA). B, The PWL to radiant heat stimulation decreased from 2 wk and became lower 4 wk after STZ injection (N = 10 for each group, **P < .01, compared with CNT group, two‐way repeated‐measures ANOVA followed by Tukey post hoc test). C, ALA at 60 and 120 mg/kg caused a dramatic increase in the PWT, whereas NS and 30 mg/kg had no significant effect on the PWT (N = 6 for each group, *P < .05, compared with Pre, Friedman ANOVA). D, ALA at 60 and 120 mg/kg caused a great increase in the PWL, whereas NS and 30 mg/kg had no significant effect on the PWL (D, N = 6 for each group, *P < .05, compared with Pre, Friedman ANOVA). E, 60 mg/kg ALA injection had no significant effect on the PWT in CNT rats (N = 6 for each group, *P* > .05, Mann‐Whitney *U* test). F, ALA at 60 mg/kg produced no influence on the PWL in CNT rats (N = 6 for each group, *P* > .05, compared with Pre, two‐sample *t* test)

Consistent with our previous studies,^8^, ^14^ a single injection of STZ induced hindpaw hypersensitivity in rats. As is shown in Figure 1A‐B, the mechanical PWT and PWL were decreased 2 and 4 weeks after STZ injection (Figure 1A, N = 10 for each group, *P < .05, **P < .01, compared with CNT group, Friedman ANOVA; Figure 1B, N = 10 for each group, **P < .01, compared with CNT group, two‐way repeated‐measures ANOVA followed by Tukey post hoc test). Two weeks after the injection of citrate buffer or STZ, the mechanical PWT was 11.48 ± 0.88 g and 8.99 ± 0.66 g, and the thermal PWL was 15.38 ± 0.38 seconds and 13.68 ± 0.38 seconds, respectively, for control (CNT) rats and diabetic (STZ) rats. Four weeks after the injection, the mechanical PWT was 11.07 ± 0.98 g and 5.60 ± 0.97 g; and the thermal PWL was 14.74 ± 0.30 seconds and 12.04 ± 0.38 seconds, respectively. We then studied the effects of ALA on hindpaw hypersensitivity in rats. Three weeks after STZ injection, ALA in different doses (30, 60, and 120 mg/kg) or normal saline (NS) was administrated to diabetic rats by intraperitoneal injection once a day lasting for 1 week. The results showed that 30 mg/kg of ALA, as well as NS, had no apparent influence on PWT and PWL. The mechanical PWT for before and after injection of 30 mg/kg dose ALA was 4.16 ± 1.02 g and 8.98 ± 1.15 g, and the thermal PWL was 10.08 ± 0.50 seconds and 10.24 ± 0.46 seconds, respectively. The mechanical PWT for before and after injection of NS was 4.87 ± 0.92 g and 3.07 ± 0.58 g, and the thermal PWL was 9.82 ± 0.28 seconds and 8.62 ± 0.49 seconds, respectively. However, there is a dramatic increase when the concentration of ALA is 60 and 120 mg/kg on the PWT and PWL (Figure 1C, N = 6 for each group, *P < .05, compared with Pre, Figure 1D, N = 6 for each group, *P < .05, compared with Pre, Friedman ANOVA). The mechanical PWT for before and after injection of 60 mg/kg dose ALA was 3.26 ± 0.84 g and 10.12 ± 0.39 g, and the thermal PWL was 9.30 ± 0.48 seconds and 12.80 ± 0.99 seconds, respectively. The mechanical PWT for before and after injection of 120 mg/kg dose ALA was 3.68 ± 0.54 g and 11.42 ± 1.09 g, and the thermal PWL was 9.50 ± 0.21 seconds and 13.38 ± 1.01 seconds, respectively. We also observed the effect of ALA at 60 mg/kg on the age‐ and sex‐matched healthy control rats. The results reflected that ALA at 60 mg/kg did not bring significant influence on the PWT and PWL in CNT rats (Figure 1E, N = 6 for each group, *P* > .05, Mann‐Whitney *U* test; Figure 1F, N = 6 for each group, *P* > .05, compared with Pre, two‐sample *t* test).

### 3.2 The excitability of DRG neurons was enhanced by STZ injection

**Figure 2:**
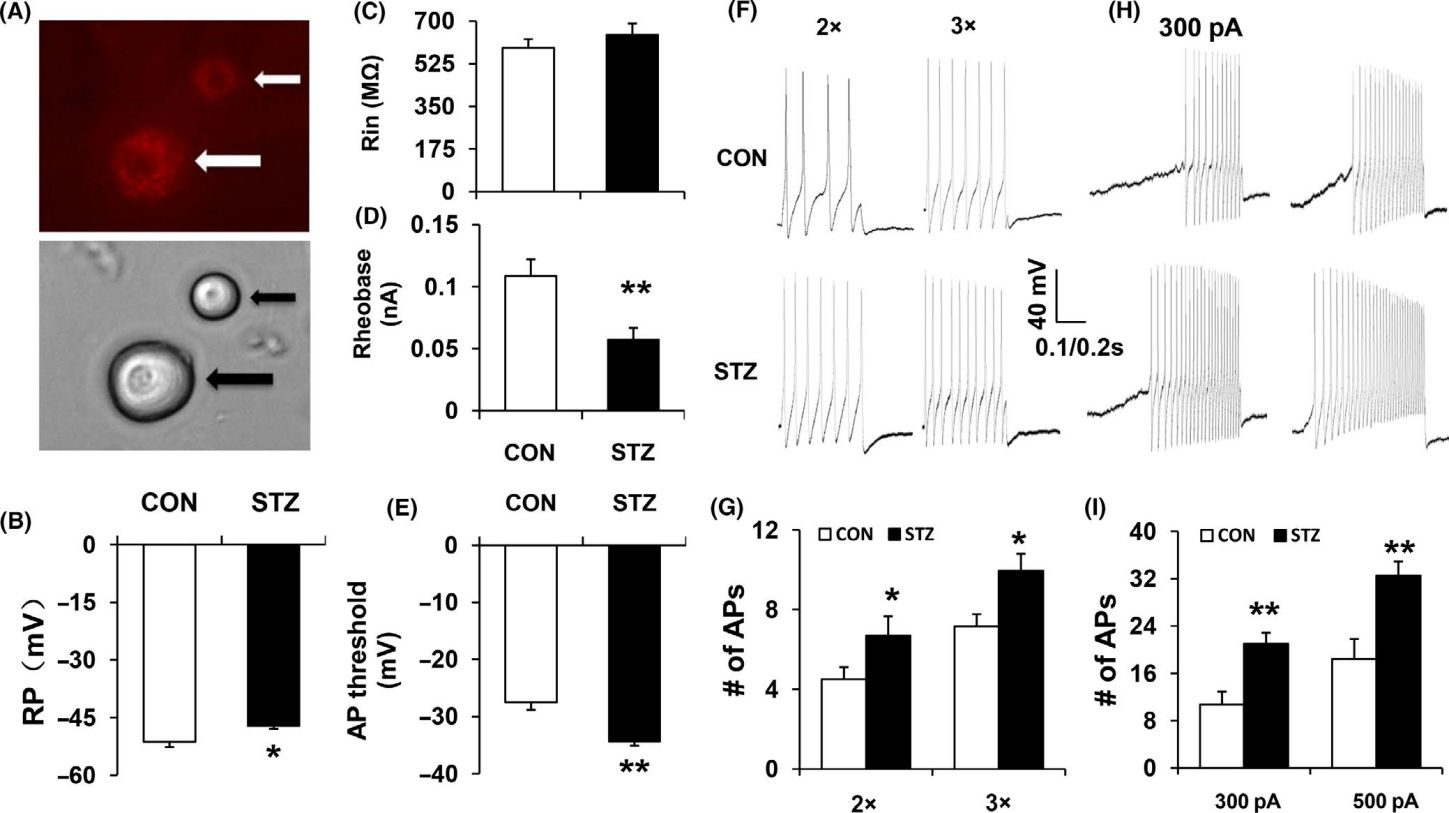
Changes in membrane properties of DRG neurons innervating the hindpaw 4 wk after STZ injection. A, Top: Arrows show examples of two DRG neurons with Dil‐labeled. Bottom: Image of the same DiI‐labeled DRG neurons. Scale bar = 50μm. Patch‐clamp recordings were performed on DiI‐labeled DRG neurons. A total of 20 DiI‐labeled neurons from control rats (CNT) and 20 DiI‐labeled neurons from diabetic rats (STZ) were recorded with current‐clamp conditions. B, STZ injection depolarized the resting membrane potential (RP) in DiI‐labeled DRG neurons (*P < .05, compared with control rats, two‐sample t test). C, STZ injection caused no remarkably change of Rin (*P* > .05, compared with control rats, Mann‐Whitney U test). D, STZ injection caused apparently reduction of rheobase (**P < .01, compared with control rats, Mann‐Whitney *U* test). E, STZ injection significantly hyperpolarized action potential (AP) threshold (**P < .01, compared with control rats, Mann‐Whitney *U* test). F, Image involved the examples of AP trances evoked by 2 × and 3 × rheobase current stimulation from CNT (top) and STZ‐injected rats (bottom). G, Bar graphs indicated that STZ injection resulted in a greatly increase on the frequencies of APs evoked by 2 × and 3 × rheobase current stimulation (*P < .05, compared with control rats, two‐sample t test). H, Image indicated the examples of APs by 300 and 500 pA ramp current injections from control and STZ‐injected rats. I, The frequencies of APs in response to 300 and 500 pA ramp current stimulation significantly increased in DRG neurons from STZ‐injected rats compared with control rats (**P < .01, compared with control rats, two‐way repeated‐measures ANOVA followed by Tukey post hoc test)

DRG neurons innervating the hindpaw were labeled by the fluorescent dye 1, 1'‐dioctadecyl‐3,3,3',3'‐tetramethylindocarbocyanine (DiI) (Figure 2A, arrow). As is shown in Table 1, we observed a significant depolarization in the resting membrane potential (RP) of DRG neurons in STZ‐induced diabetic rats (Figure 2B; n = 20 for each group,*P < .05, compared with CNT, two‐sample *t* test). The input resistance (Rin) of DRG neurons did not alter after STZ injection (Figure 2C; n = 20 for each group, *P* > .05, compared with control rats, Mann‐Whitney *U* test). Rheobase, which was the minimal injected current that evokes one AP, was much lower in the STZ group than in the CNT group (Figure 2D; n = 20 for each group, **P < .01, compared with control rats, Mann‐Whitney *U* test). Action potential (AP) threshold was hyperpolarized in the STZ group when compared with the CNT group (Figure 2E; n = 20 for each group, **P < .01, compared with control rats, Mann‐Whitney *U* test). The frequency of APs in response to two times rheobase (2×) and three times rheobase (3×) current stimulation was significantly enhanced in DRG neurons of STZ‐induced diabetic rats (Figure 2F,G; n = 20 for each group,*P < .05, two‐sample *t* test). The frequencies of APs in response to 300 and 500 pA ramp current stimulation were strongly increased in STZ‐induced diabetic rats (Figure 2H,I; n = 18 for each group, **P < .01, compared with control rats, two‐way repeated‐measures ANOVA followed by Tukey post hoc test).

**Table 1:** Membrane characteristics of hindpaw projection dorsal root ganglion neurons in control and streptozotocin‐induced diabetic rats

	CON	STZ	*P* value
Cell size (µm)	27.1 ± 0.5 (n = 20)	26.9 ± 0.5 (n = 20)	.79
Cm (pF)	29.0 ± 1.4 (n = 20)	27.6 ± 1.1 (n = 20)	.43
Rin (MΩ)	590.1 ± 35.7 (n = 20)	644.0 ± 46.4 (n = 20)	.36
RP (mV)	‐51.3 ± 1.4 (n = 20)	‐47.3 ± 0.7 (n = 20)	<.05
Rheobase (nA)	0.11 ± 0.01 (n = 20)	0.06 ± 0.01 (n = 20)	<.01
# of APs (2×)	4.5 ± 0.6 (n = 20)	6.7 ± 0.6 (n = 20)	<.05
# of APs (3×)	7.2 ± 1.0 (n = 20)	10.0 ± 0.9 (n = 20)	<.05
AP Threshold (mV)	‐27.5 ± 1.6 (n = 20)	‐34.4 ± 1.6 (n = 20)	<.01
AP Amplitude (mV)	83.5 ± 2.7 (n = 20)	83.4 ± 2.4 (n = 20)	.95
AP Overshoot (mV)	35.9 ± 2.5 (n = 20)	33.8 ± 2.4 (n = 20)	.51
AP Duration (ms)	2.9 ± 0.2 (n = 20)	2.6 ± 0.2 (n = 20)	.07
Latency (ms)	106.2 ± 23.8 (n = 20)	108 ± 18.2 (n = 20)	.35
# of APs (0.1 nA)	2.8 ± 0.9 (n = 18)	9.8 ± 1.5 (n = 18)	<.05
# of APs (0.3 nA)	10.7 ± 2.2 (n = 18)	21.0 ± 1.8 (n = 18)	<.01
# of APs (0.5 nA)	18.4 ± 3.4 (n = 18)	32.5 ± 2.4 (n = 18)	<.01
# of APs (1.0 nA)	28.5 ± 4.7 (n = 18)	43.1 ± 3.4 (n = 18)	<.01
TTFS (ms, 0.1 nA)	761.5 ± 65.24 (n = 18)	488.9 ± 66.1 (n = 18)	<.01
TTFS (ms, 0.3 nA)	483.5 ± 63.4 (n = 18)	274.3 ± 39.7 (n = 18)	<.01
TTFS (ms, 0.5 nA)	368.3 ± 60.9 (n = 18)	203.6 ± 32.9 (n = 18)	<.01
TTFS (ms, 1.0 nA)	186.5 ± 33.8 (n = 18)	107.3 ± 20.2 (n = 18)	.2

Note

Values are mean ± SEM, with sample size in parenthesis. *P*‐values were determined by two‐sample *t* test, Mann‐Whitney test or two‐way repeated‐measures ANOVA followed by Tukey post hoc test where appropriate.

Abbreviations: AP, action potential; Cm, membrane capacitance; Rin, input resistance; RP, resting membrane potential; TTFS, time to first spike.

### 3.3 The excitability of DRG neurons from diabetic rats was decreased by ALA injection

**Figure 3:**
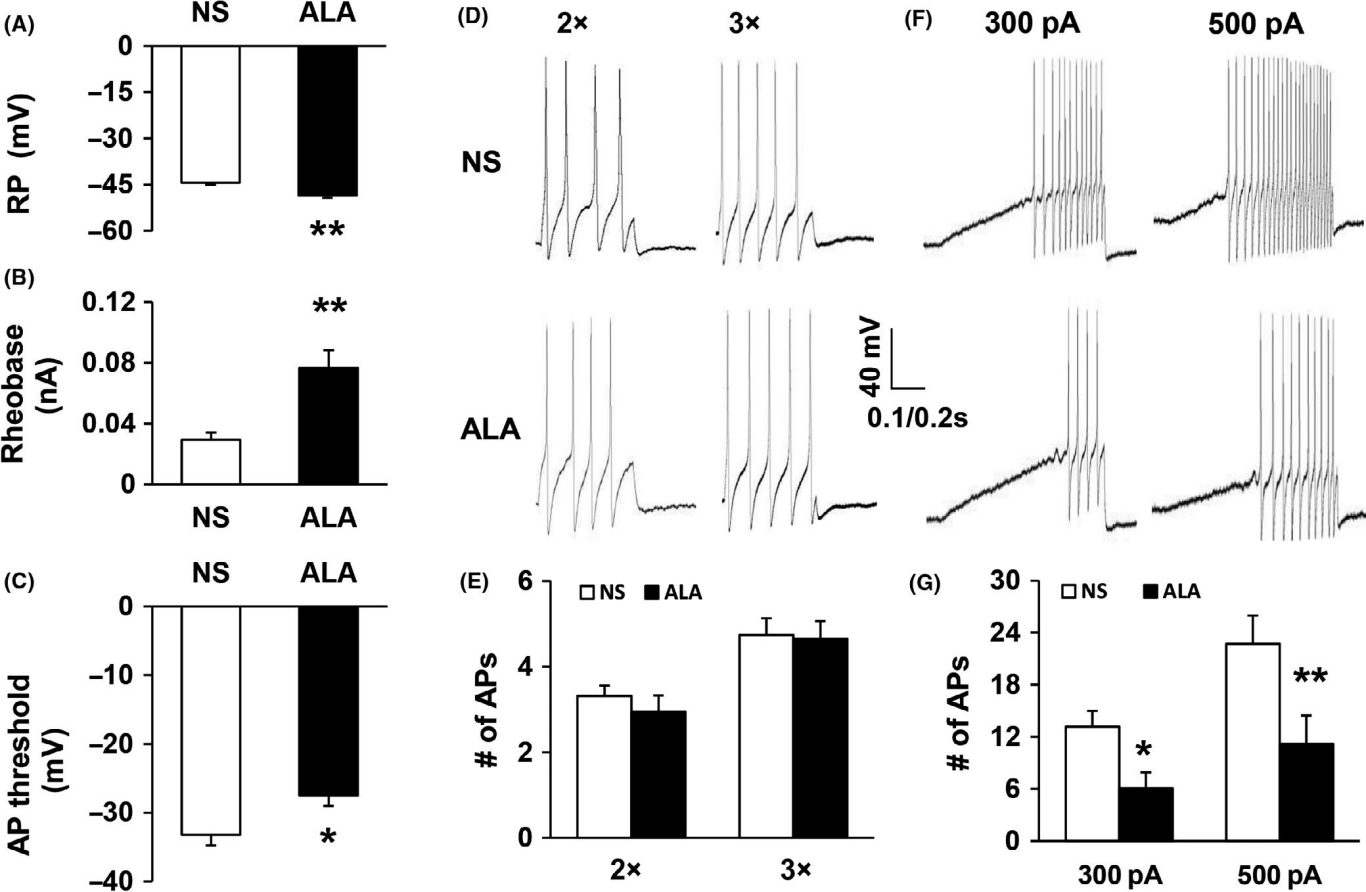
All the changes occurred in membrane properties of DRG neurons innervating the hindpaw after ALA treatment. A, The resting membrane potential (RP) was hyperpolarized in DRG neurons of diabetic rats after ALA treatment (*P < .01, compared with NS, Mann‐Whitney *U* test). B, ALA treatment caused an apparently enhancement of rheobase (*P < .01, compared with NS, Mann‐Whitney *U* test). C, ALA treatment resulted in significantly depolarization in action potential (AP) threshold (*P < .05, compared with control rats, two‐sample *t* test). D, Image indicated the instances of AP trances evoked by 2 × rheobase and 3 × rheobase current stimulation from NS (top) and ALA‐injected diabetic rats (bottom). E, Bar graphs indicated that ALA treatment caused no strong increase on the frequencies of APs evoked by 2 × rheobase and 3 × rheobase current stimulation (*P* > .05, compared with NS, Mann‐Whitney *U* test). F, Image involved the examples of APs by 300 pA and 500 pA ramp current injection from NS‐ and ALA‐treated diabetic rats. G, The frequencies of APs in response to 300 and 500 pA ramp current stimulation remarkably decreased in DRG neurons from ALA‐treated rats compared with NS rats (*P < .05, *P < .01, compared with NS, two‐way repeated‐measures ANOVA followed by Tukey post hoc test)

In this part of the experiment, we obtain DRG neurons innervating the hindpaw from the STZ‐injected diabetic rats after the injection of normal saline (NS) and alpha‐lipoic acid (ALA) once a day for consecutive 7 days, respectively (Table 2). We could observe an obvious hyperpolarization on the RP of DRG neurons innervated the hindpaw from ALA‐injected diabetic rats (Figure 3A; n = 19 for NS group, n = 17 for ALA group, **P < .01, compared with NS, Mann‐Whitney *U* test). Meanwhile, the rheobase was much higher in the ALA group than in the NS group (Figure 3B; n = 19 for NS group, n = 17 for ALA group, **P < .01, compared with NS, Mann‐Whitney *U* test). Compared with the NS group, AP threshold had a great depolarization in the ALA group (Figure 3C; n = 19 for NS group, n = 17 for ALA group, *P < .05, compared with control rats, two‐sample t test). The frequency of APs, which was in response to two times (2×) and 3 times (3×) rheobase current stimulation, had no significant reduction in DRG neurons of ALA‐induced diabetic rats (Figure 3D,E; n = 19 for NS group, n = 17 for ALA group, *P* > .05, Mann‐Whitney *U* test). However, the frequency of APs in response to 300 and 500 pA ramp current stimulation was sharply decreased in ALA‐treated diabetic rats (Figure 3F,G; n = 11 for NS group, n = 15 for ALA group, *P < .05, **P < .01, compared with NS, two‐way repeated‐measures ANOVA followed by Tukey post hoc test).

### 3.4 TRPV1 was upregulated in diabetic rats and was suppressed by ALA treatment

**Figure 4:**
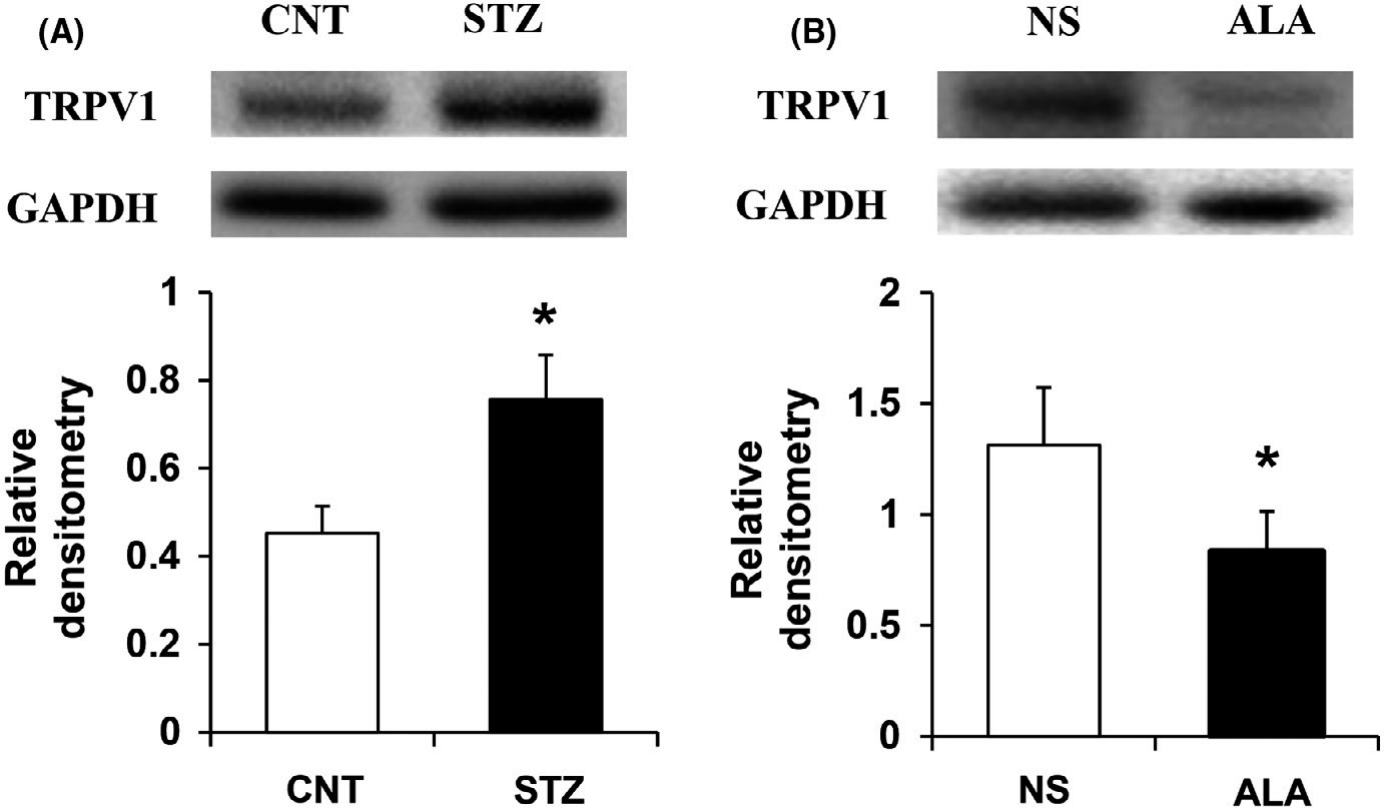
STZ injection upregulated the expression of TRPV1 in DRG, whereas ALA downregulated TRPV1 expression. A, Western blots for TRPV1 of DRGs innervating the hindpaw from control and STZ injection rats. Bar graph indicated mean density relative of glyceraldehyde‐3‐phosphate dehydrogenase (GAPDH) and TRPV1 from CNT and STZ rats. STZ injection significantly enhanced expression of TRPV1 of DRGs innervating the hindpaw (N = 4 for each group, *P < .05, compared with CNT, two‐sample t test). B, Bar graph showed mean density relative to GAPDH for TRPV1. ALA treatment strongly reduced the expression of TRPV1 (N = 4 for NS group, N = 3 for ALA group, *P < .05, compared with NS, two‐sample *t* test)

After STZ injection, the expression of TRPV1 increased apparently (Figure 4A; N = 4 for each group; *P < .05, compared with CNT, two‐sample t test). The relative density of TRPV1 was 0.45 ± 0.06 and 0.76 ± 0.10 in CNT and STZ groups, respectively. Three weeks after STZ injection, the same volume of NS and ALA at 60 mg/kg dose was i.p. injected once a day for consecutive 7 days. ALA injection suppressed TRPV1 expression at protein levels, when compared with NS (Figure 4B; N = 4 for NS group, N = 3 for ALA group; *P < .05, compared with CNT, two‐sample *t* test). The relative density of TRPV1 was 1.38 ± 0.22 and 0.68 ± 0.03 in NS and ALA groups, respectively.

**Table 2:** Membrane characteristics of hindpaw projection dorsal root ganglion neurons in streptozotocin‐induced diabetic rats treated with NS and ALA

	NS	ALA	*P* value
Cell Size (µm)	25.4 ± 0.8 (n = 19)	26.5 ± 0.7 (n = 17)	.33
Cm (pF)	26.1 ± 1.6 (n = 19)	26.9 ± 1.6 (n = 17)	.73
Rin (MΩ)	525.4 ± 32.6 (n = 19)	558.1 ± 52.2 (n = 17)	.59
RP (mV)	‐44.4 ± 0.6 (n = 19)	‐48.5 ± 0.7 (n = 17)	<.001
Rheobase (nA)	0.03 ± 0.004 (n = 19)	0.08 ± 0.01 (n = 17)	<.001
# of APs (2×)	3.3 ± 0.2 (n = 19)	2.9 ± 0.4 (n = 17)	.13
# of APs (3×)	4.7 ± 0.4 (n = 19)	4.7 ± 0.4 (n = 17)	.77
AP Threshold (mV)	‐33.2 ± 1.5 (n = 19)	‐27.5 ± 1.5 (n = 17)	<.05
AP Amplitude (mV)	83.7 ± 2.9 (n = 19)	77.5 ± 2.9 (n = 17)	.15
AP Overshoot (mV)	39.0 ± 2.2 (n = 19)	29.6 ± 2.6 (n = 17)	<.01
AP Duration (ms)	4.4 ± 0.4 (n = 19)	4.4 ± 0.4 (n = 17)	.97
Latency (ms)	129.0 ± 19.8 (n = 19)	122.4 ± 21.4 (n = 17)	.7
# of APs (0.1 nA)	6.4 ± 1.2 (n = 11)	1.9 ± 0.5 (n = 15)	.12
# of APs (0.3 nA)	13.2 ± 1.8 (n = 11)	6.1 ± 1.1 (n = 15)	<.5
# of APs (0.5 nA)	22.7 ± 3.3 (n = 11)	11.2 ± 1.8 (n = 15)	<.01
# of APs (1.0 nA)	32.5 ± 5.2 (n = 11)	16.5 ± 2.4 (n = 15)	<.01
TTFS (ms, 0.1 nA)	415.3 ± 68.4 (n = 11)	557.2 ± 97.8 (n = 15)	.34
TTFS (ms, 0.3 nA)	265.9 ± 39.4 (n = 11)	246.8 ± 65.1 (n = 15)	.06
TTFS (ms, 0.5 nA)	147.9 ± 22.4 (n = 11)	143.3 ± 36.7 (n = 15)	.29
TTFS (ms, 1.0 nA)	96.1 ± 13.1 (n = 11)	108.2 ± 37.2 (n = 15)	.46

Note

Values are mean ± SEM, with sample size in parenthesis. *P*‐values were determined by two‐sample *t* test, Mann‐Whitney test or two‐way repeated‐measures ANOVA followed by Tukey post hoc test where appropriate.

Abbreviations: AP, action potential; Cm, membrane capacitance; Rin, input resistance; RP, resting membrane potential; TTFS, time to first spike.

### 3.5 TRPV1 and NF‐κB were colocalized in DRG neurons

**Figure 5:**
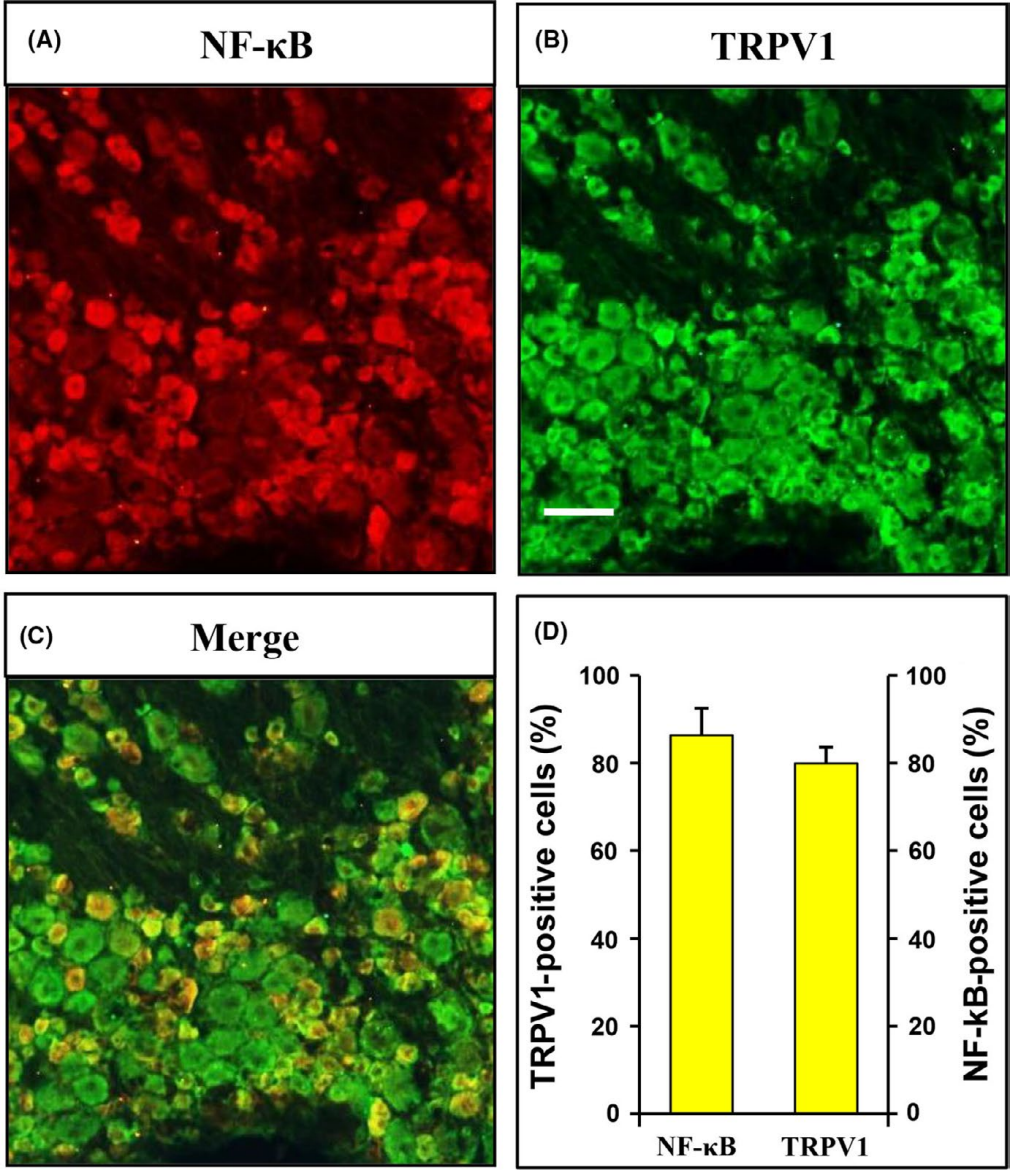
TRPV1 and NF‐κB were co‐expressed in DRG neurons. A, NF‐κB‐positive cells were shown in red. B, TRPV1‐positive cells were shown in green. C, Merge of double labeling of TRPV1 and NF‐κB. Scale bar was 50 μm. D, Quantified analysis showed the majority of TRPV1 was co‐expressed with NF‐κB‐positive DRG neurons, and the majority of NF‐κB was also co‐expressed with TRPV1‐positive DRG neurons

We first injected Dil into the plantar skin of bilateral hindpaws to retrogradely label hindpaw‐specific DRG neurons and then studied the localization of TRPV1 and NF‐κB in DRG by immunofluorescence technique. As shown in Figure 5A,B, NF‐κB‐positive cells (red, Figure 5A) and TRPV1‐positive cells (green, Figure 5B) colocalized in the DRG neurons (yellow, Figure 4C). The graph showed that the majority of TRPV1 was co‐expressed with NF‐κB in the same DRG neurons (Figure 4D, N = 3). The percentage of TRPV1‐positive cells in all NF‐κB‐positive cells was 86.38%, and the percentage of NF‐κB‐positive cells in TRPV1‐positive cells was 79.96%.

### 3.6 ALA inhibited TRPV1 expression via NF‐κB

**Figure 6:**
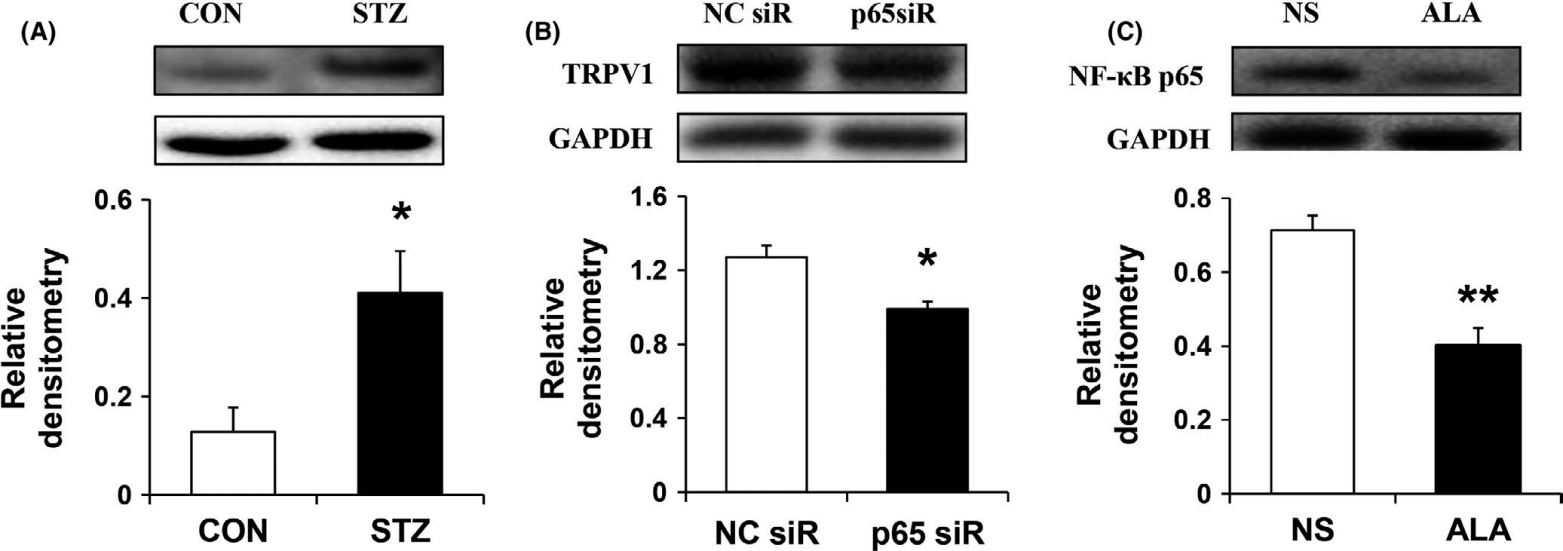
ALA inhibited TRPV1 expression via suppressing NF‐κB. A, Western blots for NF‐κB in DRGs innervating the hindpaw from control and STZ‐induced diabetic rats. Bar graph involved mean density relative of β‐actin and NF‐κB from control and STZ‐induced diabetic rats. STZ injection significantly enhanced expression of NF‐κB (N = 4 for each group; *P < .05, compared with CNT, two‐sample *t* test) in L4‐L6 DRGs. B, Western blots for TRPV1 in L4‐L6 DRGs from diabetic rats treated with p65 siRNA lentivirus and NC siRNA lentivirus, respectively. Graph showed mean density relative to GAPDH for TRPV1. The lentiviruses were intrathecally injected into rats. The expression of TRPV1 significantly reduced after p65 siRNA lentiviruses treatment compared with NC siRNA group (N = 4 for each group, *P < .05, compared with NC siR, two‐sample t test). C, Western blots for NF‐κB in L4‐L6 DRGs from diabetic rats treated with NS and ALA, respectively. Graph showed mean density relative to GAPDH for NF‐κB. ALA treatment strongly reduced the expression of NF‐κB (N = 4 for NS group, N = 3 for ALA group, **P < .01, compared with NS, two‐sample t test)

In line with our previous studies,^8^, ^14^ the results showed that NF‐κBp65 had a vital enhancement 4 weeks after STZ injection compared with control rats (Figure 6A, N = 4 for each group; *P < .05, compared with CNT, two‐sample t test). The relative density of NF‐κB was 0.13 ± 0.05 and 0.41 ± 0.05 in CNT and STZ groups, respectively. After we selectively knock down the expression of p65 with p65 siRNA lentivirus (LV‐p65 siRNA), the expression of TRPV1 downregulated correspondingly. As shown in Figure 6B, TRPV1 expression in p65 siR group was less than in NC siR group (Figure 6B, N = 4 for each group; *P < .05, compared with NC siR, two‐sample *t* test). The relative density of TRPV1 was 1.27 ± 0.07 and 0.99 ± 0.07 in NC siR and p65 siR groups, respectively. In addition, NF‐κB markedly decreased in diabetic rats after one‐week ALA treatment, when compared with NS treatment (Figure 6C, N = 4 for NS group, N = 3 for ALA group; **P < .01, compared with NS, two‐sample *t* test). The relative density of NF‐κB was 0.71 ± 0.04 and 0.40 ± 0.05 in NS and ALA groups, respectively.

## 4 DISCUSSION

Diabetic neuropathy (DN) is a common complication of diabetes and troubles many patients with long‐term diabetes. Oxidative stress is considered to be the most important pathogenesis of DN. Amounts of evidence from randomized, double‐blind, placebo‐controlled clinical trials and meta‐analyses suggest that antioxidant ALA is an effective and safe first‐line treatment because of its excellent effect in reduction of oxidative stress, improvement in nerve blood flow, nerve conduction velocity, and several other measures of nerve function.^16^ However, painful diabetic neuropathy (PDN) is thought to have its unique mechanisms, including changes in ion channel activity in peripheral and central sensory neurons, abnormal regeneration of sympathetic nerve fibers, and dysfunction of central descending inhibition systems. The therapeutic effect of antioxidative stress therapy on PDN was once not optimistic. Recently, some studies reported that ALA monotherapy or in combination with gabapentin or pregabalin could alleviate the pain symptoms of diabetic patients.^17^ Moreover, our previous studies found that ALA can reverse the high expressions of NaV1.7, NaV1.8, and P2X3 receptors in DRG neurons of STZ‐induced diabetic rats.^18^, ^19^ We suppose that ALA may alleviate neuropathic pain in diabetes by affecting the excitability of DRG neurons and the sensitization of the peripheral nervous system.

In the present study, we also applied the STZ‐induced diabetic neuropathic pain model. Consistent with our previous results,^8^, ^14^ STZ‐induced diabetic rats displayed a reduction in PWT and PWL for mechanical and thermal stimulations, as shown in Figure 1A‐B. In addition to the mechanical allodynia and thermal hyperalgesia, the excitability of hindpaw‐innervating DRG neurons labeled by the fluorescent dye DiI was significantly increased in diabetic rats, as shown in Figure 2A‐I. This result was mainly based on the observations that the resting membrane potential was depolarized, the AP threshold was hyperpolarized, the rheobase was decreased, and the frequency of APs evoked by 2 and 3 times rheobase stimulation and ramp current stimulation was increased. Combined with others’ studies,^20^, ^21^ these data suggest that the sensitization of DRG neurons is an important feature in STZ‐induced diabetic neuropathic pain in rats. It is well known that the sensitization of DRG neurons is an important mechanism of neuropathic pain. Primary sensory neurons in DRG are sensitized to environmental noxious stimuli, continually transmitting signals to the central nervous system, eventually forming pain sensation. Some agents relieve pain by affecting the sensitization of DRG neurons.^20^ We next studied the effects of ALA on the mechanical allodynia, thermal hyperalgesia, and DRG neuronal excitability in diabetic rats. The results showed that ALA treatment could increase the PWT and PWL and reduce the excitability of DRG neurons. These data initially confirmed our hypothesis that ALA may alleviate neuropathic pain in diabetes by affecting the excitability of hindpaw‐specific DRG neurons.

How does ALA affect neuronal excitability? Growing evidences have shown that changes in neuronal excitability are mainly related to the plasticity of sodium, potassium, and calcium channels in neurons.^22^, ^23^, ^24^ In addition, ligand‐gated channels are also considered to play an important role in neuronal sensitization. For example, TRPV1 and purinergic P2X receptor played important roles in diabetes‐induced pain sensitivity, through increasing current density and enhancing signal transmission.^8^, ^25^ Recently, we reported that ALA could reduce the expression and function of P2X3 in DRG neurons from diabetic rats.^18^ Actually, TRPV1 is also a representative ligand‐gated channel that plays a role in diabetic neuropathic pain. So, in the present study, we determined its expression and the result showed that TRPV1 expression in the hindpaw‐innervating DRGs from diabetic rats was greatly increased 4 weeks after STZ injection. Then does ALA affect the expression of TRPV1 similar to the effect on P2X3 expression? The result was miraculous that ALA treatment could also significantly reduce TRPV1 expression. Combined with our previous findings, these data suggested that ALA might act on multiple ligand‐gated channels through certain pathways, and further affect the excitability of DRG neurons.

NF‐κB is an important nuclear transcription factor in cells and involved in inflammation, immune, and stress responses. Others and our studies reported NF‐κB also participates in chronic pain.^8^, ^26^ ALA could inhibit the NF‐κB signaling pathway.^27^ We speculated that ALA might affect TRPV1 expression by inhibiting NF‐κB pathway. We first used immunofluorescence assay to study the distribution of TRPV1 and NF‐κB. Because studies have shown that NF‐κB and TRPV1 are mainly distributed in the pain‐related small and medium neurons,^28^, ^29^ we only studied whether they are co‐expressed in the same neurons in this study. The results showed that NF‐κB and TRPV1 colocated in most of the same neurons. Also, consistent with our previous findings, NF‐κB is activated in DRG from diabetic rats. The results showed that p65, the main active ingredient of NF‐κB, was upregulated in hindpaw‐innervating DRG in diabetic rats. To further investigate the regulation of NF‐κB on TRPV1, we applied p65 siRNA lentivirus to specifically knock down the expression of p65 and intrathecal injection of this virus could effectively reduce the expression of p65 in hindpaw‐innervating DRG.^8^ Intrathecal injection of LV‐p65 siRNA significantly decreased TRPV1 expression in diabetic rats. Selective knockdown of p65 expression and TRPV1 expression decreased correspondingly, suggesting that p65 has a regulatory effect on TRPV1. So far, we found that NF‐κB is indeed an important regulator of diabetic neuropathic pain. It not only regulates the expression of CBS, one of the endogenous H_2_S synthetase but also affects the ligand‐gated ion channels P2X3 and TRPV1. We supposed that ALA might reduce the effects of P2X3 and TRPV1 by inhibiting NF‐κB. In the present study, we determined the p65 expression after ALA treatment. The results showed that ALA did decrease the expression of p65. NF‐κB is an important mediator of the analgesic effect for ALA treatment.

However, we did not further study how p65 regulates TRPV1 expression. From bioinformatics prediction (http://www.gene‐regulation.com/pub/programs/alibaba2/index.html), there are 3 transcription‐binding sites for p65 in the TRPV1 promoter region (data not shown), suggesting that p65 may also affect the expression of TRPV1 through transcriptional regulation. Besides, it may work through other unknown mechanisms, such as indirectly regulating the expression of TRPV1 by affecting other regulatory factors.

## 5 CONCLUSION

Our findings demonstrated for the first time that ALA might alleviate neuropathic pain and reduce the excitability of DRG neurons in diabetes by suppressing TRPV1 expression via inhibiting NF‐κB activation.

## CONFLICT OF INTEREST

The authors declare no conflict of interest.

## AUTHOR CONTRIBUTIONS

B.‐YZ and Y.‐L. Z. researched and analyzed the data and wrote the article. QS, P.‐AZ, and X.‐X. W. researched and analyzed the data. JH reviewed and edited the article. H.‐H. Z. designed and supervised the study and edited the article.
